# Exploring the Effect of Metformin to Lower Thyroid-Stimulating Hormone in Euthyroid and Hypothyroid Type-2 Diabetic Patients

**DOI:** 10.7759/cureus.13283

**Published:** 2021-02-11

**Authors:** Sundus Mariyum Haroon, Khurshid Khan, Muhammad Maqsood, Sadaf Iqbal, Muhammad Aleem, Tahir Ullah Khan

**Affiliations:** 1 Internal Medicine, Jinnah Hospital, Lahore, PAK; 2 Jinnah Allama Iqbal Institute of Diabetes and Endocrinology (JAIDE), Jinnah Hospital, Lahore, PAK; 3 Medicine, Fatima Memorial College of Medicine and Dentistry, Lahore, PAK; 4 Internal Medicine, University of Lahore, Lahore, PAK

**Keywords:** type-2 diabetes mellitus, metformin, hypothyroid, euthyroid

## Abstract

Introduction

Metformin is one of the safest, first-line oral hypoglycemic agents used in type-2 diabetes mellitus patients. This study aims to study the effect of metformin on thyroid-stimulating hormone (TSH) in hypothyroid and euthyroid individuals, as both these diseases have an increased prevalence and coexistence.

Method

This hospital-based study was conducted in Jinnah Allama Iqbal Institute of Diabetes and Endocrinology (JAIDE), Allama Iqbal Medical College/Jinnah Hospital Lahore, Pakistan, from October 2019 to April 2020. One hundred and sixty type-2 diabetic participants, aged 25-60 years and meeting the inclusion criteria were enrolled in the study after informed consent. They were divided into two groups, the hypothyroid group who were already on levothyroxine therapy and had a stable TSH in the normal range, and a euthyroid group who had no thyroid dysfunction. Both the groups were started on metformin therapy for the control of type-2 diabetes mellitus and followed for six months. Their blood samples for TSH and free thyroid hormone (fT4) were drawn both prior to and after the study period.

Results

Out of the 160 type-2 diabetic patients, TSH levels showed a significant reduction in the hypothyroid patients (2.33 ± 0.70, p < 0.001) with no significant changes in the euthyroid patients (3.87 ± 0.40, p = 0.206) following six months of metformin therapy. However, there was no significant difference in the fT4 levels in either of the groups.

Conclusion

Metformin has the effect of significantly lowering TSH levels in hypothyroid individuals. However, no such effect was observed in euthyroid patients.

## Introduction

Hypothyroidism and diabetes mellitus (DM) both being chronic diseases have a long-lasting effect on cardiovascular morbidity and mortality. Hypothyroidism further increases cardiovascular risk factors, such as hypertension and hyperlipidemia [1]. There is an increased prevalence of hypothyroidism in type-2 diabetic patients. Thyroid disorder in the general Indian population is estimated to be 10% [2], while its prevalence in diabetic population has been estimated at 24.8% [3]. In China, the prevalence of hypothyroidism among type-2 DM inpatients was 6.8% [4]. However, according to a study carried out in Khyber Pakhtunkhwa, Pakistan, the prevalence of hypothyroidism in the general population was significantly high and estimated to be 31.8% [5]. This implies to the wide range of demographic variables in different populations.

Metformin is one of the most commonly used oral hypoglycemic agents for the treatment of type-2 DM. This biguanide derivative acts as an insulin sensitizer in the liver by decreasing hepatic gluconeogenesis and increases the uptake of glucose in the skeletal muscles by activating AMP-activated protein kinase (AMPK) [6]. It has relatively few side effects and no clinically relevant drug interaction. Hence, it is commonly regarded as a safe drug [7]. Gastrointestinal side effects (nausea, diarrhea, abdominal pain, dyspepsia) occur in about 28% people, in which discontinuation is rarely considered (<2%). The risk of lactic acidosis is rare and mostly occurs in the presence of other comorbidities.

Several hypotheses have been put forward to explain the action of metformin on thyroid-stimulating hormone (TSH). Metformin can cause activation of the TSH receptor by changing the quantity and affinity of the thyroid hormone receptors thereby enhancing the effects of the thyroid hormones in the pituitary gland [8].

Metformin has been found to cross the blood-brain barrier (BBB) in a study conducted on rats, with its highest concentration found in the pituitary. It suppresses AMPK activity in the hypothalamus despite activating it in the periphery and, therefore, enhances the effect of thyroid hormones in the pituitary which results in the suppression of TSH [9].

Additionally, metformin can inhibit the growth of thyroid cells as well as thyroid cancer cells by altering insulin/IGF1 and mTOR pathways. Hence patients treated with metformin have a reduced thyroid volume and a lesser risk of incident goiter [10]. This is in contrast to hyperinsulinemia, commonly associated with type-2 DM, in which individuals have a greater thyroid volume and a higher incidence of thyroid nodules and cancers [11].

Few intriguing studies have shown that metformin has a suppressive effect on TSH in hypothyroid patients, the first reported by Vigersky et al. in 2006 [12]. Since then, certain prospective and retrospective studies have revealed conflicting and contradictory results [13].

A recent study has indicated that the effect of metformin on hypothalamic-pituitary-thyroid axis activity may be determined by sex. It was predominantly in women that metformin decreased serum TSH levels [14].

Furthermore, metformin has not reported to enhance the gastrointestinal absorption of levothyroxine (l-T4) in patients with hypothyroidism. A clinical study indicated that serum thyroid hormone levels remained unchanged in response to metformin irrespective of whether thyroxin replacement was given or not [15].

Metformin being one the most commonly used drugs for type-2 DM in Pakistan, its role in altering TSH levels amounts to paramount importance in patients with coexisting DM and hypothyroidism. As the prevalence of coexisting DM and hypothyroidism is high in the Pakistani population and the effect of metformin on TSH has not been evaluated previously, this study, therefore, aims to determine the effect of metformin in hypothyroid patients already on levothyroxine replacement as well as in euthyroid patients.

## Materials and methods

This interventional study was conducted at Jinnah Allama Iqbal Institute of Diabetes and Endocrinology (JAIDE) in Jinnah Hospital, for a period of six months from October 2019 to April 2020. The study was conducted following the principles of good clinical practice, as laid down in the Declaration of Helsinki, after approval of its synopsis by the ethical review board of Allama Iqbal Medical College, Lahore. One hundred and sixty type-2 diabetic patients, between 25 and 60 years of age, either newly diagnosed or previously diagnosed in accordance with American Diabetes Association criteria were recruited for the study who were not on metformin therapy. Written informed consent was obtained from each participant. The patients were divided into two categories, diagnosed hypothyroid patients, already on levothyroxine therapy with TSH levels in the normal reference range and euthyroid patients, having no thyroid dysfunction.

Diabetics with comorbidities, newly diagnosed and untreated hypothyroid patients and hyperthyroid patients previously treated with either radioactive Iodine or surgery and now on replacement levothyroxine therapy, were excluded from the study. Demographic information regarding height, weight, and gender were included in the study. Body mass index (BMI) was calculated as body weight (in kg) divided by the square of body height (in meters). Patients were inquired regarding diabetic and hypothyroid symptoms, previous tests or screening carried out, medications ever used for the treatment of either condition, the presence of comorbidities and family history of such complaints. Venous blood samples for HbA1c, and thyroid function tests (TSH and fT4) were drawn. Thyroid function tests were analyzed using a chemiluminescent immunoassay TSH3-Ultra (ADVIA Centaur® CP Immunoassay System) (normal range of TSH (0.35-5.5mIU/L) and fT4 (11.5-23pg/mL). Patients were started on metformin therapy and monitored for six months. HbA1c was monitored three times monthly and thyroid function tests prior to and after six-month study period were recorded and compared.

Sample selection was done with the help of non-probability purposive sampling technique. Sample size calculation was done with the formula and information: n = Z^2^ P(1-P)/d^2^, where n = sample size, Z = Level of confidence (95%), P = expected prevalence or proportion = 10%, d = margin of error = 5%, and sample size (n) = 160. Patients were selected with the help of predefined sample selection criteria. The data were entered in Statistical Package for Social Sciences (SPSS) version 20.0 (IBM Statistics Incorporated, Chicago, IL, USA) and analyzed. Continuous variables including age, weight, height, BMI, HbA1c, TSH levels and fT4 levels were analyzed via descriptive statistics and were presented as means and SDs. T-test and Chi-square were applied as appropriate. P-value of less than 0.05 meant that the difference between the groups is significant and the null hypothesis is void.

## Results

Out of the 160 diabetic patients, 100 (62.5%) were euthyroid and 60 (37.5%) were hypothyroid. Mean age of diabetic patients was 45.2 ± 7.8 (range: 25-60) years. Overall, 84 (52.5%) were females and 76 (47.5%) were males. Mean HbA1c was 6.0% ± 1.4, which ranged from 5.5% to 8.0%.

All hypothyroid patients had initial TSH and fT4 levels, prior to metformin therapy, in the normal reference ranges. The baseline TSH level in the hypothyroid group ranged from 1.4 to 4.5mIU/L whereas in the euthyroid group it ranged from 2.5 to 4.5mIU/L. Following six months on metformin, the hypothyroid group had TSH in the range of 1.0-3.2mIU/L (mean ± SD; 2.33 ± 0.70), whereas the euthyroid group had TSH in the range of 3.0-4.3mIU/L (mean ± SD; 3.87 ± 0.40). Post metformin, the TSH levels showed a significant reduction in the hypothyroid patients (p < 0.001) with no significant changes in the euthyroid patients (p = 0.206). The baseline fT4 level in the hypothyroid group ranged from 11.5 to 20.2pg/mL and in the euthyroid group it ranged from 11.5 to 22.2pg/mL. On the other hand, fT4 levels failed to show any significant changes in both the groups after six months of metformin therapy (p > 0.05). The mean values of serum TSH and fT4 levels, at baseline and post-metformin therapy, in both hypothyroid and euthyroid patients are displayed in Table 1.

**Table 1:** TSH and fT4 levels among hypothyroid and euthyroid patients TSH, thyroid-stimulating hormone; fT4, free thyroid hormone.

TSH (mIU/L)	Hypothyroid patients (n = 60)	Euthyroid patients (n = 100)
Baseline	3.66 ± 0.84	3.95 ± 0.60
After six-month on metformin	2.33 ± 0.70	3.87 ± 0.40
P-value	< 0.001	0.206
fT4 (pg/mL)		
Baseline	14.96 ± 2.07	15.85 ± 1.86
After six-month on etformin	14.54 ± 2.06	15.70 ± 1.67
P-value	0.103	0.146

## Discussion

The results of the present study suggest that in our population metformin treatment has a suppressive effect on the circulating TSH concentration in hypothyroid patients on levothyroxine therapy but has no significant effect on euthyroid patients. Following six months of therapy with metformin, the TSH levels showed a significant reduction in the hypothyroid patient group (p < 0.001) with no significant changes in the euthyroid patient group (p = 0.206).

There have been some contradictory results in different studies regarding the action of metformin on TSH [16]. Certain retrospective studies have suggested a peculiar “buffer effect” of metformin on the TSH concentration according to which a slight but significant increase of TSH level was observed when TSH was in the lower-normal range, whereas a slight decrease was observed when it was in the upper-normal range. This was observed in euthyroid diabetic patients [17]. In another prospective study conducted on prediabetic patients, similar suppressive effects of metformin were observed only in those patients with a basal high-normal TSH concentration [18]. A prospective study by the Cappelli group in 2009 observed the effect of metformin on 54 euthyroid diabetic patients which suggested that metformin did not have a significant effect on serum TSH level. In their second paper in 2012 which analyzed 393 type-2 DM patients, there was a metformin-lowering TSH effect on patients having high -normal basal serum TSH levels as well as on hypothyroid patients [19]. In a longitudinal cohort analysis conducted in Canada, Laurent Azoulay, PhD, of McGill University in Montreal reported that there was a 55% increased risk of hypothyroid patients with type-2 diabetes having suppressed TSH levels while on metformin therapy, compared with hypothyroid diabetic patients who were taking a sulfonylurea [20].

In contrast, the Pakistani population has shown TSH to fall to a spuriously low subnormal level proving a challenge for the treating clinician in the decision to alter the already stable levothyroxine dosage. Therefore, the decision to treat coexisting diabetes in hypothyroid patients with metformin has its challenges of monitoring the thyroid status with greater avidity, adjacent to its positive impact on the suppression of TSH.

## Conclusions

The effect of metformin in significantly lowering TSH levels in hypothyroid patients on levothyroxine therapy without causing any changes in the circulating hormone levels has raised questions regarding the complexity of monitoring such patients. This study emphasizes the importance of taking into consideration that metformin initiation falsely lowers circulating TSH levels within a few months and may compel the treating physician/endocrinologist to bring about unnecessary dosage adjustments in levothyroxine. If the clinician is aware of this effect of metformin, then major dosage readjustments may be successfully prevented. Therefore, the results of this study indicate that not only should there be a re-evaluation of the thyroid-pituitary axis within 6-12 months of initiating metformin in diabetic patients having concomitant hypothyroidism, but the dose of levothyroxine may also not be changed unless TSH falls below the lower reference range with a raised fT4 suggesting iatrogenic hyperthyroidism.
